# Advances on Food-Derived Peptidic Antioxidants—A Review

**DOI:** 10.3390/antiox9090799

**Published:** 2020-08-27

**Authors:** Mingfei Pan, Kaixin Liu, Jingying Yang, Shengmiao Liu, Shan Wang, Shuo Wang

**Affiliations:** 1 State Key Laboratory of Food Nutrition and Safety, Tianjin University of Science and Technology, Tianjin 300457, China; panmf2012@tust.edu.cn (M.P.); Liukx2019@163.com (K.L.); yangjy0823@126.com (J.Y.); lsm20000711@outlook.com (S.L.); wshan0929niu@163.com (S.W.); 2 Key Laboratory of Food Nutrition and Safety, Ministry of Education of China, Tianjin University of Science and Technology, Tianjin 300457, China

**Keywords:** food-derived peptidic antioxidants, purification, characterization, performance evaluation

## Abstract

The oxidation process is considered to be the main reason behind human aging, human degenerative diseases and food quality degradation. Food-derived peptidic antioxidants (PAs) have wide sources and great activity, and have broad application prospects in removing excess reactive oxygen species in the body, anti-aging and preventing and treating diseases related to oxidative stress. On the other hand, PAs are expected to inhibit the lipid peroxidation of foods and increase the stability of the food system in the food industry. However, the production pathways and action mechanism of food-derived PAs are diverse, which makes it is difficult to evaluate the performance of PAs which is why the commercial application of PAs is still in its infancy. This article focuses on reviewing the preparation, purification, and characterization methods of food-derived PAs, and expounds the latest progress in performance evaluation and potential applications, in order to provide an effective reference for subsequent related research of PAs.

## 1. Introduction

Since the theory of free radicals was proposed, more and more studies have proved that the degenerative changes in the aging process are related to the production of reactive oxygen species (ROS) such as superoxide anion radical (O_2_^•−^), hydroxyl radical (^•^OH), lipid radical (ROO^•^) and reactive nitrogen species such as nitrogen oxide (NO^•^) during cellular metabolism [1,2,3]. Generally, free radicals in the body are continuously generated, and there also exists a defense system, composed of endogenous antioxidants (glutathione (GSH), etc.) and antioxidant enzymes (superoxide dismutase (SOD), glutathione peroxidase (GSH-Px), peroxidase, etc.), to maintain the normal metabolic balance of ROS and further protect the body from free radical damage [4,5]. However, when the body ages or is in a bad environment, free radicals are excessively produced or removed slowly, causing oxidative stress [6]. Oxidative stress can cause oxidation of the substances that constitute cell tissues such as lipids, carbohydrates, proteins, and DNA, resulting in oxidative damage such as denaturation, cross-linking, and breakage [7,8]. A large number of studies have demonstrated that many organ dysfunctions or tissue lesions such as heart disease, stroke, arteriosclerosis, diabetes, and cancer are related to the increased content of ROS in the body [9,10]. Therefore, it is necessary to seek exogenous antioxidants that can maintain the balance of free radical metabolism in the body together with the endogenous antioxidant system to adjust and improve human physiological functions, so as to achieve the purpose of preventing and treating chronic diseases [11,12]. Additionally, unfavorable factors such as heat, light-sensitive transition metals, metal proteins and radiation can lead to food lipid peroxidation, that cause changes in food quality such as color, smell, tissue structure and nutritional components and result in a decline in food quality and even the production of harmful substances [13]. Therefore, it is necessary to add antioxidants in the production process to maintain food quality (especially for meat products) [14]. Synthetic antioxidants with strong antioxidant effects such as 2,6-Di-tert-butyl-4-methylphenol (BHT) and butylhydroxyanisole (BHA) are restricted or prohibited due to the potential teratogenic and carcinogenic effects on the human body [15].

**Figure 1:**
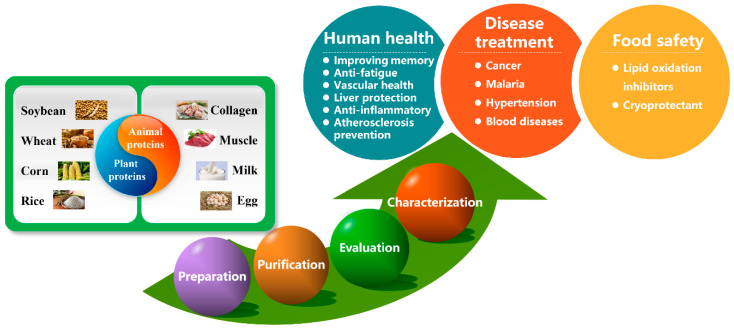
Protein sources, research methods and potential applications of peptidic antioxidants.

Recently, the development of high-efficiency and safe antioxidants from natural products, especially foods, has become one research hotpot and has attracted widespread attention from researchers. Except for some well-known natural antioxidants (such as vitamins, bioflavonoids, carotenoids, proteins, amino acids, etc.), peptides also have the same antioxidant mechanism [16,17]. Compared to amino acids and macromolecular proteins, peptides with a structure between them have extremely biological diversity and more significant antioxidant properties. In addition, peptidic antioxidants (PAs) can be ingested safely, and usually possess other biological activities such as antibacterial, anti-hypertensive and cholesterol reduction [18,19,20,21]. Based on the above advantages, PAs have become potential raw materials for the development of new functional foods, health products, and food additives. Some PAs are naturally present in foods. However, due to the low content and limited sources, the extraction operation of such peptides is complicated and costly, which makes it difficult for industrial production and commercial application. Studies have shown that peptide sequences with antioxidant activity are often found in food proteins (edible proteins, and waste proteins in food processing), and they need to be released through certain technologies (Figure 1) [22,23]. Due to the wide sources and abundant content of precursor proteins, these types of PAs can greatly improve the economic benefits of the food industry and promote environmental protection, with a wide range of practical applications (Figure 1) [24,25].

This article reviews the latest advances of PAs in foods and food processing by-products, especially the methods of preparation, purification, and characterization, and also summarizes the structure–activity relationship (SAR) of PAs derived from food proteins. Additionally, due to the complexity of the antioxidant mechanism of bioactive peptides, this article comprehensively analyzes the main methods for PA performance evaluation and summarizes its application prospects in food-related fields in order to provide a certain reference for the subsequent research of PAs.

## 2. Preparation of PAs Derived from Food Proteins

The protein in foods is an important source of PAs, and the bioactive peptides can be released by suitable degradation means from the proteins [26]. At present, the methods for producing PAs through degrading proteins mainly include enzymatic hydrolysis, gastrointestinal (GI) digestion, and microbial fermentation and other food processing [27].

### 2.1. Enzymatic Hydrolysis

Enzymatic hydrolysis is a method for preparing PAs by using endogenous or exogenous proteases to degrade food proteins [28]. Because the conditions of the hydrolysis process are relatively mild and easy to control, enzymolysis is currently the most frequently used strategy in preparing PAs, developing new PAs and studying the SAR of PAs [29,30]. Table 1 illustrates the PAs prepared by enzymatic hydrolysis using edible animal and plant proteins as raw materials in recent years. The SAR indicates that the PA properties are mainly related to the molecular weight (Mw), the composition of amino acids and hydrophobicity [31,32]. The types of food proteins and enzymes directly affect the structure of PAs. Soybean protein [33,34,35], wheat protein [36], corn protein [37], rice protein [38] of plant origin and muscle protein [39,40], collagen [41], milk protein [42], egg protein [43] of animal origin have been used for the preparation of PAs. Additionally, the parameters of time, added amount, temperature and pH may affect the degree of hydrolysis (DH) of the substrate proteins, which further affect the size and amino acid composition of the obtained peptides [44,45]. Due to the different targets and cleavage methods of enzymes, the kinds and properties of enzymes are crucial to the preparation of PAs by hydrolysis [46]. Compared to the endogenous enzymes, exogenous proteases have higher hydrolysis efficiency and are controllable for hydrolyzed products [47]. In the early days, proteases were selected by comparing the antioxidant properties of hydrolysates obtained under optimal conditions through preliminary biochemical experiments [48]. Wattanasiritham et al. used Trypsin and Papain to hydrolyze rice bran protein (RBP), in which the RBP hydrolyzed by Trypsin had the highest antioxidant activity [49]. Although this strategy is blind and needs a cumbersome experimental process, it is still the main way to develop new PAs and improve the system of SAR. With the deepening of SAR research, databases related to PAs have been gradually constructed, and the quantitative SAR (QSAR) bioinformatics have also been gradually applied to proteases selection and exploration of new PAs [50,51]. Esfandi et al. compared the effect of different enzymes on the extraction and hydrolysis to oat bran proteins by peptide analysis method and antioxidant activity measurement [52]. The results showed that Viscozyme-proteins hydrolyzed with Papain showed stronger antioxidant capacity. The effect of 21 different enzymes and enzyme combinations on the release of potential PAs in major yolk protein of sea urchin Strongylocentrotus nudus was analyzed by in silico analysis [53]. From the obtained results, the Proteinase *K*, Papain and GI tract enzymes have the best hydrolysis effect (the number of peptides effectively released are 20, 16 and 13–15, respectively). The use of bioinformatics can transform the tedious, time-consuming and high-cost process of exploring new PAs into a simple, systematic, and designable one, which has wide application prospects [54].

At present, the enzymes commonly used in the preparation of food-derived PAs mainly include microbial-derived industrial proteases (Alcalase, Flavourzyme, Protamex and Neutral Proteases), plant-derived proteases (Papain) and animal-derived proteases (Pepsin and Trypsin) [75] (Table 1). Some proteases for the hydrolysis of specific food proteins have been applied for the development of PAs. For example, collagenase can specifically hydrolyze collagen to obtain peptides with high antioxidant activity [76,77]. In addition, some newly developed proteases have also been applied to hydrolyze proteins to prepare PAs. Metalloproteases and Serine proteases have been respectively prepared from fungus *Eupenicillium javanicum* and *Myceliophthora thermophila* [78]. These two proteases were used to digest egg albumin, casein, and whey protein, and new PAs were separated and purified from the three protein hydrolysates. Since the hydrolysis of proteases is carried out at specific sites, single enzymes have a relatively small range of hydrolysis, and complex hydrolysis of two or more proteases will often achieve more effective results [79]. The multi-enzyme hydrolysis process is mainly divided into biphase sequential enzymolysis mode, two-step enzymolysis mode and their combined method [80]. Zhang et al. used a mixed enzyme (Papain:Protamex = 1:1) and Flavorzyme to hydrolyze egg extract in sea cucumber (*Apostichopus japonicus*) step by step, and gradually purified the hydrolysate [62]. Finally, a pure PA (Mw: about 30 kDa) with a high ^•^OH scavenging capacity of 89.82 U/mL was obtained.

In industrial production, the preparation of PAs by the traditional enzymatic hydrolysis method has many shortcomings such as the one-time use of enzymes, unstable performance of products from a different batch, long production cycle and high labor intensity [81,82]. Many efforts have been made in continuous hydrolysis modes such as the use of an enzyme membrane reactor (EMR) and the immobilized enzymes for the above shortcomings [83]. EMR is a type of reaction equipment that uses a membrane with an appropriate pore size to separate enzymes and substrates from the products, and allows the products to continuously pass through the membrane, achieving the purpose of simultaneous progress of enzymatic hydrolysis and separation [84,85]. Compared with traditional methods, EMR has many advantages such as continuous operation, reaction–separation coupling, good enzyme stability and reusability, effective enrichment, which improves the catalytic efficiency and product yield [86]. Tanaskovic et al. studied the effect of continuous EMR (ultrafiltration (UF) module (10 kDa)) on Alcalase digestion of egg white protein [87]. The results showed that the continuous EMR can improve and strengthen the enzyme reaction process and enhance *2*,*2*-Diphenyl-*1*-picrylhydrazyl (DPPH) and *1,1*-diphenyl-*2*-picryl-hydrazyl (ABTS) radical scavenge activity, and ferric reducing antioxidant power (FRAP) of the hydrolysates. The immobilized enzyme technology refers to the establishment of the enzyme in a specific position to maintain its long-term catalytic ability, reusability and controllability of the reaction [88]. Neto et al. used bovine casein as raw material and applied the protease from *Penicillium aurantiogriseum* immobilization on polyaniline-coated magnetic nanoparticles to prepare PAs [89]. Compared with non-immobilized Trypsin, covalently immobilized Trypsin on functionalized graphene oxide nanosheets exhibited significantly enhanced thermal stability, pH resistance and activity retention ability, and improved the free radical scavenging activity of the hydrolysate [90].

Appropriate pretreatment technology can promote the release of PAs during enzymatic hydrolysis of food proteins (Table 2) [91,92,93]. Besides the traditional heat treatment, new treatment strategies such as microwave [94], ultrasound [95], high pressure (HP) [96] and pulsed electric field (PEF) [97] have also been applied to improve the efficiency of enzymatic hydrolysis in the preparation of PAs. The microwave treatment has the advantages of promoting the efficiency of the reaction, strong selectivity, easy operation, fewer by-products, high yield and easy purification of the product [98]. Ketnawa et al. compared the effect of microwave pretreatment and microwave-assisted treatment on the hydrolysis of fish frame protein by Alcalase [99]. The research demonstrated that microwave treatment can significantly improve protein solubility, protein recovery, DH, and ABTS radical scavenging activity. The antioxidant activity of fish peptides prepared by microwave pretreatment (5 min) followed by conventional enzymatic hydrolysis (2–10 min) is superior to other treatment methods. The ultrasound can form the microbubbles in the liquid medium, and its rupture is accompanied by the release of energy, which can affect protein rearrangements and aggregate formation, thereby improving the biological activity of the hydrolysate [100,101,102,103]. The effect of ultrasonic and heat treatment on the enzymatic hydrolysis of barley beer waste protein by Alcalase showed that the ultrasonic pretreatment (50 kHz) can increase the antioxidant activity of protein hydrolysates and promote the release of PAs [104]. Under optimal ultrasonic conditions, low-frequency ultrasound-assisted enzymatic hydrolysis can effectively improve DH and conversion rate of corn protein and the free radical scavenging activity of the hydrolysate, and promote the formation of short-chain peptides (200–3000 Da) containing hydrophobic amino acids [105]. HP pretreatment/auxiliary treatment can increase the hydrolysis rate and DH of the substrate protein, promote the release of low Mw peptides and essential amino acids, and improve the antioxidant activity of the hydrolysate [106], which has been applied to promote the proteolysis process of legume [107,108] and peanut protein [109]. Additionally, high hydrostatic pressure (HHP) also has the ability to improve the efficiency of enzymatic hydrolysis [110]. HHP auxiliary treatment can improve the enzymatic hydrolysis efficiency of Corolase PP, reduce the surface hydrophobicity of the hydrolysate, and increase the production of small peptides [111,112]. PEF is a non-thermal processing technology that is used to sterilize, inactivate enzymes, extract functional active substances, and improve the nutritional value of foods. Therefore, PEF is often used to increase the activity of PAs after food proteolysis and separation of small PAs [113,114,115]. Studies have shown that PEF does not destroy the stability of PAs, but affects the secondary structures (α-helix, β-turn and random coil) of peptides and reduces zeta potential [116,117]. In addition, radio frequency pretreatment, high-energy electron beam, and protease co-extrusion technology have also been shown to be beneficial for improving the enzymatic hydrolysis efficiency and activity of PAs [118,119,120].

Chemical hydrolysis based on acid-base hydrolysis has also been used to prepare PAs. However, due to the destructive effect of this method on amino acids, the great changes in the composition of hydrolysate, and the high production cost, this method has been gradually replaced by enzymatic hydrolysis [121]. Protease enzymolysis has the advantages of mild conditions, specific degradation sites, fast, controllable, high repeatability, and high safety of the product, which is one hotspot in the research of PAs. As mentioned above, new technologies are devoted to develop new PAs more efficiently and accurately and to solve problems in the enzymatic hydrolysis process, such as reducing production costs and maintaining the activity of PAs. However, the current hydrolysis process is usually accompanied by the production of some bitter peptides, which need further research for its removal.

### 2.2. Microbial Fermentation and Other Food Processing

Microbial fermentation is one traditional way of producing and preserving foods, which can increase the nutritional and health value of foods [122,123]. Due to the action of microorganisms and endogenous proteolytic enzymes, PAs can be produced during the fermentation process [124,125]. Many kinds of fermented milk have been proven to be an important source of bioactive peptides [126,127,128]. Four peptides with high antioxidant activity in both chemistry and cell model evaluations have been purified and identified from the pasteurized milk fermented with *Lactobacillus acidophilus* NCFM^®^, *Lactobacillus delbrueckii* subs. *bulgaricus* and *Streptococcus thermophilus* [129]. Fermented meat products can also be used as a source of PAs [130]. By mixing and fermenting pork, koji and salt, fermented meat sauce can produce antioxidant tripeptide (Gln-Tyr-Pro) with the ^•^OH scavenging activity greater than 90% through the proteolysis process [131]. A dipeptide (Trp-Pro) with high ABTS radical scavenging activity (*EC*_50_ 17.52 ± 0.46 μM) was identified from Thai traditional fermented shrimp paste [132]. The purified component of fermented fish (pekasam) has strong ABTS radical scavenging activity (*IC*_50_ = 0.636 mg/mL), and two new PAs (AIPPHPYP, IAEVFLITDPK) were identified [133]. Compared with the enzymatic hydrolysis method, the fermentation method can simultaneously carry out the enzyme production and enzymatic hydrolysis processes using the microorganism, which reduces the cost. During the fermentation process, the exopeptidase produced by the microorganism has a modification effect on the small peptide end, which not only avoids the generation of bitterness but imparts the natural fermented flavor to the foods. Based on the above, fermented foods are potential sources of PAs. Additionally, microbial fermentation plays an important role in preparing PAs from the by-products of the food industry [134,135]. It has demonstrated that the type of inoculated microorganisms and the maturation time affect the concentration and size of peptides [136]. In the fermentation process, *Bacillus subtilis* [137,138], *lactic acid bacteria* (LAB) [139,140,141] and *fungi* [142] can release many different kinds of peptides with antioxidant activity. The three small peptides extracted from solid-state fermented sesame meal have demonstrated to have high DPPH radical and ^•^OH scavenging ability and can reduce the level of malondialdehyde (MDA) in the serum and liver of mice, and improve the liver SOD and GSH-Px activity [143]. At present, there are many studies focused on the exploration and analysis of bioactive components in the microbial fermentation broth, especially antioxidant components [144,145]. However, the research on the technology and mechanism of microbial fermentation to produce PAs still need to be explored.

Protein hydrolysis occurring in other food processing processes will naturally produce large amounts of peptides. This phenomenon is often caused by endogenous enzymes in mature foods [146]. In some processed foods, such as dried shrimp, fish sauce, tuna, ham, pot meat, non-dairy creamer, white bread and noodles, there is a peptide cyclo (His-Pro) with antioxidant activity, which can protect the body from oxidative stress and prevent GSH depletion caused by glutamate, rotenone, paraquat and beta-amyloid treatment [147,148]. From Jinhua ham, one peptidic antioxidant (PA) (GKFNV) was purified and identified with strong DPPH radical scavenging activity [149]. Spanish dry-cured ham is considered a good source of natural bioactive peptides [150]. The peptide with an Mw of 464.17 Da (SNAAC) extracted from Spanish dry-cured ham is a good antioxidant [151]. Another PA (AEEEYPDL) identified from Spanish dry-cured ham was found to have good heat and salt resistance [152].

## 3. Purification and Identification of PAs

### 3.1. Purification of PAs

Protein-derived PAs exist at low concentrations in complex systems containing different amino acid compositions. Therefore, suitable separation and purification methods are needed to enrich peptide fragments with high antioxidant activity. At present, the commonly used purification strategies for PAs mainly include membrane and chromatographic separation technology based on the changes of Mw, charge and polarity/hydrophobicity [153,154]. The membrane separation technology, containing microfiltration, UF and nanofiltration, is cheap and easy to operate, often applied for the initial purification [155]. Among them, UF is currently the most widely used method for separating PAs from food protein hydrolysates (Table 1). The centrifugal UF filters with different Mw cut-off membranes (100, 50, 30, 10 and 3 kDa) have been used in the separation of hydrolysates of Pinto bean protein isolate [59]. The obtained peptides with Mw < 3 kDa were demonstrated to have the highest free radical scavenging and FRAP activity. This is because the peptides with lower Mw are more likely to react with lipid radicals, thereby reducing free radical-mediated lipid peroxidation reaction. As mentioned earlier, the continuous integration of EMR in the enzymatic hydrolysis reaction and UF membrane separation can improve the enzymatic hydrolysis process and prepare peptides with satisfactory antioxidant activity. The traditional membrane separation driven by pressure has low selectivity for peptides with similar Mw, and can easily cause membrane contamination at HP. Electrodialysis with filter membrane (EDFM) has increased the electric field as an additional driving force based on the traditional pressure-driven UF, significantly improving the membrane migration efficiency and selectivity [156]. The separation of PAs by EDFM depends on the charge (using the different potential as a driving force for migration) and the Mw (the screening effect of the filter) of the peptides. Suwal et al. used a two-step continuous EDFM process to directly separate rainbow trout box protein hydrolysate and obtain the cationic and anionic peptides with high antioxidant activity [157]. EDFM is considered to be a more environmentally friendly technology because it does not require the use of solvents in the separation process. However, compared with other membrane separation techniques, the production efficiency of EDFM is lower, warranting further research.

The commonly used chromatography techniques in the separation of PAs include gel filtration chromatography (GFC), ion-exchange chromatography (IEC), reversed-phase high-performance liquid chromatography (RP-HPLC), preparative HPLC (prep-HPLC) and affinity chromatography, among others (Table 1). GFC is an efficient, simple, and gentle separation method based on the difference of Mw, which is mostly used to separate and purify water-soluble polymer substances and is mainly applied in the first stage of separation of PAs [66]. IEC has significant advantages in the separation of different amounts of positive and negative charged peptides [158,159]. RP-HPLC is a method of ion exchange using polar media as the main stationary phase, which separates peptides based on Mw and hydrophobicity [160,161]. In practical applications, it is usually necessary to combine multiple separation methods to separate the target product, and to avoid the incompatibility of the mobile phase in the multi-dimensional system. Jang et al. combined the UF, prep-HPLC and RP-HPLC to separate and purify sandfish protein hydrolysates and screened PAs in each stage by in vitro chemical evaluation method, where it was demonstrated that the purified polypeptide has higher antioxidant activity than protein hydrolysates [162].

Additionally, surface plasmon resonance (SPR) technology can monitor the interaction between molecules in real-time and offer a label-free detection, which is also considered in screening PAs [163]. Canabady-Rochelle et al. developed an SPR strategy for screening metal-chelating peptides in hydrolysates, which can detect the presence of metal-chelating peptides in hydrolysate faster before starting the separation stage, and has great potential for industrial application [164].

### 3.2. PAs Identification and SAR

The identification and characterization are important parts of analyzing the SAR of peptides, which is helpful to verify the reliability of the antioxidant capacity of the screened peptides. Electrophoresis analysis technology, especially sodium dodecyl sulfate polyacrylamide gel electrophoresis, is commonly used to evaluate the Mw distribution of food proteins or hydrolysates [165,166]. Tandem mass spectrometry (MS) technology has become an effective method for analyzing Mw and amino acid sequences of PAs [60,61]. Fourier transform infrared spectroscopy (FT-IR) can be used to study the secondary structure of peptides [167]. The re-synthesis of peptides is a common method to verify the antioxidant capacity of peptides and analyze the SAR [168]. A typical example is the use of nano liquid chromatography-electrospray ionization-tandem MS to characterize seven new peptide sequences with antioxidant activity from the purified components of sesame protein hydrolysate [71]. Seven peptides were re-synthesized to verify the antioxidant activity of the identified peptides and nine kinds of SYPTECRMR derived peptides were designed and synthesized to study the SAR of the peptide with the strongest antioxidant activity, and then the QSAR of SYPTECRMR was figured out by a comparative molecular field analysis (CoMFA) model. The results show that the active sites of SYPTECRMR are located in the fragment ECRMR and seem to be related to Cys or Met residues. Moreover, the amino acid sequence SYPT and steric hindrance, electrostatic interaction and other factors will also affect the antioxidant activity of SYPTECRMR. As can be seen above, the study of the SAR can provide a theoretical basis for each link of peptide preparation, which is also supplemented during the preparation process of peptides.

The molecular structure (Mw, amino acid composition, amino acid sequence and molecular conformation) and hydrophobicity of peptides are considered to be closely related to its antioxidant activity [169,170,171]. Bioactive peptides containing 2–20 amino acid residues and with an Mw in the range of 200–3000 Da are considered to have good antioxidant activity [172,173]. Peptides with lower Mw can interact with radicals more effectively, and it is easier to exert antioxidant capacity through the intestinal barrier in vivo [174]. Dipeptides and tripeptides are considered to be highly potential PAs because they can be absorbed intact from the intestinal lumen into the bloodstream and then produce biological effects at the tissue level [175]. Amino acid composition and specific amino acid sequence are the important factors affecting the antioxidant activity of peptides [176,177]. In short, peptides with strong antioxidant activity tend to have a higher proportion of hydrophobic amino acid residues [59]. Because hydrophobic amino acid residues, especially those at the end of the peptide chain, can enhance the solubility of PAs in fats and oils, their aliphatic hydrocarbon side chains can interact with fat molecules, thereby delaying or blocking lipid transition oxidation reaction chain to protect the integrity of lipid system and membrane [178]. The hydrophobicity of the peptides also makes it easier to enter the hydrophobic target organs, thereby exerting antioxidant effects [179]. The side-chain carboxyl group of the acidic amino acid has a hydrogen-donating effect, which can chelate metal ions to weaken the radical chain reaction and achieve the antioxidant effect. Antioxidant amino acid residues such as Tyr, Met, His, Lys, Trp, and Cys are often present in polypeptides with strong antioxidant activity. The imidazole group of His is related to its metal chelation, hydrogen supply and lipid peroxidation capabilities [180]. Cysteine containing thiol can directly interact with radicals, and it has an important contribution to the antioxidant activity of peptides. Due to the specific interaction and relative spatial structure between the amino acids in the amino acid sequence of the peptide, the antioxidant activity of a single amino acid is significantly lower than that of the peptide [181,182].

Since the structure of the peptide and its mechanism of antioxidant action are extremely complex, some researchers tried to summarize the relationship between the structure of peptides and antioxidant activity in order to evaluate the potential of various food proteins as PAs precursors, guiding for the selection of proteolytic enzymes and the design and synthesis of PAs [183,184]. QSAR and in silico bioinformatics methods are effective strategies to achieve these functions [185,186]. Huang et al. have combined proteomics technology with BIOPEP analysis to prove that the myosin heavy chain of tilapia processing by-products is a good precursor for PAs [187]. When using bioinformatics to prepare PAs, the update and combination of peptide databases are also important. Deng et al. applied model population analysis to establish a QSAR model on two datasets containing antioxidant tripeptides (FTC and FRAP) [188]. This three-dimensional QSAR model is constructed through CoMFA and comparative similarity index analysis, and can be used to guide the combination design and virtual screening of new peptides [189]. Leung et al. used Proteinase K to simulate digestion of rye secalin in silico, and used computational analysis based on density functional theory to identify two tripeptides (CQV and QCA) containing cysteine [190]. In silico methods have also been applied to in vivo function prediction of PAs. Alcalase and Trypsin were used to hydrolyze Atlantic sea cucumber, and peptide fractions (<2 kDa) with high antioxidant activity in vitro were separated using UF method [191]. The main antioxidant amino acids of the two peptides and their molecular interaction with myeloperoxidase (MPO) were further analyzed using in silico methods. The results show that Alcalase can produce more peptides with both antioxidant amino acids and potential MPO inhibitory activity (35.4%). A representative peptide sequence TEFHLL produced by Alcalase has a strong molecular interaction with the active site of MPO, which is predicted to have the ability to inhibit oxidative stress in the body.

## 4. Strategies for Performance Evaluation of PAs

Although a lot of research has been performed on PAs, the action mechanism of these kinds of antioxidants has not been fully revealed. Radical scavenging efficacy, metal ion chelation and quenching singlet oxygen are considered to be the main action mechanisms of PAs [168]. Currently, the evaluation of PA performance mainly includes in vitro chemical evaluation, in vitro biological evaluation and in vivo evaluation [192]. 

### 4.1. In Vitro Chemical Evaluation

Due to the characteristics of easy operation, high efficiency, low toxicity, good reproducibility and short experimental period, in vitro chemical evaluation has become the basic method for screening PAs and quantitatively determining the antioxidant activity of peptides. PAs usually have a complex structure and action principle, resulting in the difficulty of establishing a unified evaluation index [193]. According to different free radical scavenging reaction mechanisms, in vitro chemical models can be divided into the methods based on hydrogen atom transfer (HAT) and electron transfer (ET) [194]. At present, the evaluation methods for in vitro antioxidant activity mainly include DPPH radical scavenging ability, ABTS radical scavenging ability, oxygen radical absorbance capacity (ORAC), and FRAP (Table 1). In addition, evaluation methods based on metal ion (Cu^2+^, Fe^2+^, etc.) chelating ability [195,196] and anti-lipid peroxidation ability (thiobarbituric acid reactive substance (TBARS) method, ferric thiocyanate method (FTC), etc.) [197] have also been applied to in vitro chemical models. Studies have found that the antioxidant capacity of the same antioxidant may be different by using evaluation methods based on different mechanisms. For example, the zein hydrolysate obtained by the two-step enzymatic hydrolysis method (Alcalase and Flavourzyme) has higher scavenging values of DPPH radical, ^•^OH, O_2_^•−^ and reducing power, but the Fe^2+^ chelating ability is lower than other enzymatic hydrolysis methods [198]. In addition, the chemical analysis process is easily affected by the detection environment. In particular, the assays of FRAP, TEAC, and Folin–Ciocalteu reagent for evaluating PAs need to be performed under acidic, neutral, and alkaline conditions, respectively [193]. The DPPH assays based on the principle of ET is also susceptible to pH, and DPPH radicals are only soluble in organic solvents and are not suitable for evaluating water-soluble antioxidants. Therefore, in the evaluation of in vitro activity, an appropriate evaluation scheme should be selected as comprehensively as possible or according to the antioxidant indicators that the experiment focuses on. Vasquez-Villanueva et al. applied the inhibition of the peroxidation of linoleic acid as indicators to evaluate the antioxidant activity of peach kernel protein hydrolysate and peptides at each separation stage by DPPH radical assay, ABTS radical assay, ^•^OH assay, FRAP, and finally identified 18 peptides with antioxidant activity [199]. Tovar-Perez et al. evaluated the antioxidant activity of glutelin hydrolysate from cocoa (Theobroma cacao L.) seed and peptides by DPPH, ABTS and ORAC, and purified the peptides with high DPPH and ABTS radical scavenging ability (*EC*_50_ = 237.48 and 19.29 μg/mL, respectively) [200]. It is worth noting that the model system of bulk oil, oil-in-water emulsion and muscle food can provide the expected physical and chemical environment of foods, which is an important means to evaluate the antioxidant effect of PAs in the food matrix [201].

### 4.2. In Vitro Biological Evaluation

In vitro biological evaluation, that is, introducing a ROS-induced system (H_2_O_2_, lipophilic tert-butyl hydroperoxide (*t*-BHP), and *2*-azobis-(*2*-amidinopropane) dihydrochloride (AAPH) etc.) into a biological subcellular or animal tissue homogenate system to simulate oxygen stress damage in the body, and measuring some oxidative and non-oxidative indicators to reflect the functional activity of PAs [202]. Since the cells of humans and other organisms are often exposed to different oxidants, the cell model is closer to the environment in the organism. An appropriate in vitro biological model can provide a simple and inexpensive method for evaluating the bio-availability of PAs [203,204]. Cells of target organs (such as the liver, brain, or muscles) that are often exposed to oxidative stress are generally selected as cells in vitro. Different model cells were selected according to the application purpose of the antioxidants, so as to evaluate the capacity of antioxidants to inhibit a specific oxidative stress response in the organism [205]. Currently, Erythrocytes, Human hepatoma (HepG2) cells, Caco-2 cell monolayer, Pheochromocytoma (PC-12) cells, Human colon adenocarcinoma (HT-29) cells, Human hepatocyte-derived (C3A) cells, Human leukemia T (Jurkat) cells, Human umbilical vein endothelial cells (HUVECs), etc., have been used in the evaluation of various antioxidants [206,207]. The evaluation indicators of PAs in cell models mainly include the promotion of endogenous enzymes and non-enzymatic antioxidants (SOD, catalase (CAT), GSH-Px and GSH) and the inhibition of oxidation products (ROS, MDA, oxidized glutathione (GSSG)) (Table 3). H_2_O_2_-induced Caco-2 and HT-29 cell models show that the penta-peptide (NRYHE) derived from chickpea protein hydrolysate can up-regulate the activity of antioxidant enzymes (CAT, glutathione reductase (GR) and GSH-Px) in cells, and it was observed that peptide treatment elevated the expression of Nrf2 mRNA and several relative genes NQO1, HO-1, γ-GCS regulated by Nrf2 compared to the positive control [208]. Yi et al. studied the effect of soybean peptides on H_2_O_2_-induced oxidative stress in HepG2 cells [209]. The results showed that soybean peptides inhibited the production of H_2_O_2_-induced ROS, MDA and GSSG in HepG2 cells, prevented the reduction of GSH and up-regulated the activity of cellular antioxidant enzymes (SOD, CAT and GSH-Px), which are expected to protect and regulate the body under oxidative stress. Wang et al. evaluated the changes of antioxidant activity of cooked eggs in a simulated human GI digestion model in vitro, and used the level of ROS in rat aortic vascular smooth muscle cells (VSMC) measured by dihydroethidium staining as an evaluation indicator [210]. The purified three peptides (DSTRTQ, ESKPV and DVYSF) with antioxidant activity have the potential to regulate vascular function. Cellular antioxidant activity (CAA) is a quantitative analysis method to measure the ability of target compounds to quench peroxy radical in HepG2 cells, which provide evidence of the comparison of antioxidant capacity of different peptides (Table 3). The protective effect of cells under oxidative stress is also used in the evaluation of PAs, usually by *3*-(*4*,*5*-dimethylthiazol-*2*-yl)-*2*,*5*-diphenyltetrazolium bromide (MTT) colorimetric assay. Several studies have evaluated the viability of cell models exposed to PAs without oxidative stress treatment. These experiments are used to verify whether the PAs studied are cytotoxic and provide the concentration of non-cytotoxic peptides for subsequent experiments. Using ORAC as an evaluation target, the PA CCCCSVQK was purified and identified from water-soluble protein extracts of Hanwoo beef [211]. Subsequently, the MTT method was applied to determine its effect on the proliferation of human colorectal carcinoma cells (HCT116). The results showed that CCCCSVQK dose-dependently inhibited the growth of HCT116 cells, and the maximum inhibition rate (25.24%) was obtained at 250 μg/mL. Peptides extracted from hazelnut protein hydrolysate can inhibit ROS synthesis in HUVECs, and have strong antioxidant and cytoprotective effects on Ang-induced oxidative damage, which can be used as an antioxidant in the food and pharmaceutical industries [63].

Due to the complexity of the human digestive system and internal environment, the evaluation of PAs should also consider its stability in GI digestion, cell penetration, and the action in the biological environment. At the same time, because the human body may produce similar peptides when digesting the same protein, new PAs released from food proteins through simulated digestion may be more physiologically significant [229,230,231]. The Pepsin–Pancreatin system is commonly used to simulate GI digestion in vitro, which is often used to evaluate the stability of PAs in GI [232]. The experiment of the Caco-2 cell model demonstrated that the transmembrane transport mode of wheat germ peptide (Mw < 1 kDa) is mainly passive transport, which can exert an effective antioxidant effect through intestinal epithelium [233]. Three different cases of hydrophobic casein peptides were sequentially processed through simulated GI digestion and Caco-2 cell model, and the peptide nitrogen was used to evaluate the bioavailability (BA) and remaining peptide content [234]. The results showed that the treated highly hydrophobic peptides had excellent BA and certain residual antioxidant activity, but had poor stability in GI digestion, and two GI-resistant peptides (NTVP and IV) were identified from this component.

### 4.3. In Vivo Evaluation 

The in vivo evaluation of antioxidants mainly includes the indirect determination of the degree of protection of bioactivity substances against DNA oxidative damage, lipid peroxidation, protein oxidative damage, and mitochondrial oxidative damage based on the determination of oxidative stress biomarkers, and the direct measurement of the antioxidant level of the antioxidant defense system [235]. Currently, only a few studies have evaluated the effect of food-derived PAs in animal models. In the model, the animal was fed the tested substance, and the antioxidant activity of the test substance in the organisms was evaluated by comparing the experimental group and the control. Khaled et al. verified that Sardine protein hydrolysates (SPHs) have high DPPH free radical scavenging activity and metal-chelating activity through in vitro chemical experiments, and conducted a series of animal experiments on them [236,237]. From the results, SPH treatment can reduce the concentration of MDA and increase the activity of antioxidant enzymes (SOD, GSH-Px, CAT) and high-density lipoprotein cholesterol.

Active peptides can inhibit or block lipid membranes peroxidation caused by excessive accumulation of free radicals, protect protein and nucleic acid structures in cells from oxidative damage, thereby improving antioxidant capacity and inhibiting the occurrence of oxidative fatigue. Ding et al. found that jellyfish collagen hydrolysate improved the anti-fatigue ability of mice and increased the activity of SOD and GSH-Px in mice [238]. The decline of exercise endurance is the most direct and objective indicator of fatigue. Guo et al. prepared sea-horse peptides by enzymatic hydrolysis that the DPPH radical scavenging rate is 71.89 ± 1.50% and ^•^OH scavenging rate is 75.53 ± 0.98% [239]. Furthermore, the team evaluated the anti-fatigue activity of sea-horse peptides in mice through the swimming exhaustion experiment according to the changes in blood glucose, blood lactic acid, serum urea nitrogen and liver glycogen content in mice before and after exercise. The results show that sea-horse peptides have anti-fatigue activity and its anti-fatigue effect is dose-dependent.

Generally, the in vivo experiment is more sensitive and closer to the actual system of the organism, but it has the disadvantages of long experiment time, high cost and cumbersome process. This is why the evaluation of the antioxidants usually carried out in the order in vitro to in vivo, and from the chemical environment to the biological environment. Meanwhile, the toxicity and dose–response of antioxidant peptides should be considered to ensure the authenticity and reliability of the results.

## 5. Potential Application of Food-Derived PAs 

### 5.1. Functional Ingredients to Stabilize Food Quality

Since mixed peptides or pure peptides can reduce oxidative changes during food processing or storage, they are expected to be added as functional ingredients to food systems [240]. The protective effect of PAs on the food matrix is mainly reflected in the inhibition of lipid oxidation of high-fat foods (such as food emulsions, meats, sauces, beverages), thereby delaying the loss of food nutrients and suppressing the production of harmful substances [241,242]. Studies have shown that certain PAs derived from foods (such as squid [243], mussels [244], oysters [15], shrimp muscle [245]) have the same or better ability to inhibit lipid peroxidation as the fat-soluble antioxidant α-tocopherol. In the linoleic acid model system, the tripeptide (WPP) isolated from the clam protein hydrolysate showed lipid peroxidation reduction ability similar to GSH [246]. In the sardine minced meat model system, the squid protein hydrolysate prepared by Papain has similar lipid oxidation inhibition ability as ascorbic acid [247]. Zein hydrolysate has been proven to effectively inhibit lipid oxidation, reduce the production of hydrogen peroxide and TBARS, and significantly improve the oxidative stability of model oils [248]. Moreover, this hydrolysate has no negative effect on the quality of the emulsion, and may become an effective antioxidant in food emulsion [249]. QITEGEDGGG (Caragana seed protein source) can effectively inhibit the oxidation of linoleic acid (inhibition ratio: 60.37%), delay the auto-oxidation rate of walnut oil, and produce a synergistic effect with tertiary butylhydroquinone (TBHQ) and vitamin C [250]. It is worth noting that the antioxidant properties of pure peptides have been verified by re-synthetic peptides. Milk-derived protein hydrolysates and peptides can be used to prevent lipid oxidation in muscle foods [251,252]. Casein calcium peptide (2.0%) added to beef paste homogenate can inhibit about 70% of the lipid oxidation in the homogenate, which helps to prevent the generation of odors in meat products, thereby extending the shelf life [253]. The crude peptide extract from lamb ham significantly reduces the content of thiobarbituric acid reactive substances and cross-linking of proteins in lamb cake, and also improves the color, texture and microbial stability of the product [254]. The protein hydrolysates and peptides derived from marine biological such as Kirin gelatin skin [255], Amur sturgeon skin gelatin [256], Heilongjiang fish skin [257], silver carp [258,259], shellfish [260], can be used as bifunctional ingredients (antioxidants and cryoprotectants) to delay the lipid oxidation and protein denaturation of the seafood products [261]. Gelatin hydrolysates prepared from cuttlefish skin (0.5 mg/g) can delay lipid oxidation of turkey sausage at 4 °C for up to 10 days [262]. After dipping with the hydrolysis of shrimp waste, the whole crocodile fish skin can maintain the color at 4 °C for 10 days [263]. The protein hydrolysate processed by Savinase or Protamex by-product of eel can be added to the meat emulsion as a preservative to inhibit the growth of microorganisms within 11 days at 4 °C [264]. The gelatin water-soluble chitosan film containing squid Maillard peptide can effectively preserve the fresh bluefin tuna and prolong the storage period to 8 days at 4 ± 1 °C [265]. Compared with protein hydrolysates and mixed peptides, the preparation process of pure peptides is more complicated and the cost is relatively high, resulting in less research on the maintenance of food quality. Therefore, the application of PAs as functional components must fully consider their cost. In addition, the protein hydrolysis process or the selection of specific peptides may cause changes in their composition and structure, and may produce other adverse substances that affect food quality. Therefore, before a PA is commercialized, it is necessary to evaluate its stability during food processing and storage and the potential safety or sensory problems it may cause [266].

### 5.2. Human Health Promotion and Disease Treatment

PAs have broad application prospects in promoting human health and preventing and treating diseases related to oxidative damage [267]. Some dietary supplements or skincare products that use PAs (such as GSH, carnosine, and anserine) as the main functional ingredients have been commercialized. However, most of the PAs extracted from food proteins are still in the experimental stage. As described in Section 4.2 and Section 4.3, some cell models and animal experiments have shown that PAs have a significant protective effect on cells under oxidative stress. PAs have the effects of anti-fatigue, improving memory and vascular health [268], protecting the liver [269,270], reducing ROS-related pro-inflammatory reactions [221,271] and preventing atherosclerosis [272,273], which provides a basis for their application in nutritious foods, functional foods, dietary supplements and other fields [274]. Cuttlefish-purified peptides can obviously inhibit the oxidation process of linoleic acid, effectively protect the DNA damage induced by ^•^OH radicals, and have no toxic effect to cells at higher concentrations [159]. In addition, some studies have confirmed that PAs have certain potential in the treatment of cancer [275], malaria [276], hypertension [277,278] and blood diseases [224], and are expected to become therapeutic drugs. Bioactive peptides derived from rapeseed protein (LY, RALP, GHS) can significantly inhibit the secretion of NO, il-6 and tumor necrosis factor stimulated by lipopolysaccharide, and then repair the damage of spontaneously hypertensive cells caused by oxidative stress, which has potential application value in protecting the body from oxidative and inflammatory damage [279]. At present, due to the lack of animal model experiments and human clinical trials, the research progress of antioxidant peptides in terms of bioavailability is minimal, which limits their commercialization process. On the other hand, in the application process of the food matrix, the influence of food ingredients and processing conditions on the bioavailability of PAs, as well as the production cost, biological activity, efficacy and safety of peptide-containing foods should be considered [280,281,282]. Before the development of large-scale foods containing PAs, it must be confirmed that the addition of these peptides will not cause negative effects and still retain their antioxidant activity.

## 6. Conclusions

PAs have considerable potential application value in human health promotion, disease treatment and food preservation. At present, the preparation, performance evaluation and commercial production of food protein-derived PAs have received extensive attention from researchers, which has greatly promoted the development of the field of natural antioxidants. However, due to the immature research in various fields of PAs, further development is needed. Enzymatic hydrolysis is still the main way to produce PAs from food proteins. The introduction of bioinformatics technology is expected to gradually develop the preparation of natural PAs into a predictable and controllable process. In addition, the combination of in silico and biochemical experiments is expected to become a promising strategy for developing and modifying food protein-derived PAs. Meanwhile, due to the advantages of unique food flavor and reduced production costs, microbial fermentation and other food processing methods have become indispensable means for the development of PAs, but its related mechanism of action still needs further research. The role of PAs in the prevention and treatment of oxidative stress-related diseases has been controversial. Animal experiments on bioavailability and food safety of PAs are still relatively few. Therefore, the actual effects and side effects of PAs should be further studied before clinical trials. In recent years, using food processing by-products as raw materials for the production of PAs has become a hot spot for researchers and related companies, which can effectively reduce production costs and environmental pressure. In addition, it is of great practical significance to effectively optimize and improve the processes of hydrolysis, separation and purification in industrial production to improve production efficiency.

## Figures and Tables

**Table 1:** Enzymes used for the preparation of food protein hydrolysate, the methods of purification, and the evaluation of antioxidant capacity ^1^.

Source	Enzyme and Purification Methods	In Vitro Chemical Evaluation	IC_50_ or Scavenging Activity ^2^	Amino Acid Sequence or Molecular Weight	Ref
Egg white protein powder	Alcalase; UF (30, 10, 1 kDa), GFC (Sephadex), RP-HPLC	Reducing power assay DPPH radical scavenging activity ABTS radical scavenging activity ORAC assay	ABTS (92.21 ± 0.5% at 5 mg/mL) ORAC (1238.56 ± 0.6 μmol TE/g) DPPH (FFGFN IC_50_ = 80 mM; MPDAHL IC_50_ = 60 mM)	DHTKE, FFGFN, MPDAHL	[55]
Duck (*Anas platyrhynchos*) breasts	Protamex; UF (30, 10 kDa), GFC (Sephadex G-25), IEC	DPPH radical scavenging activity ^•^OH scavenging activity Fe^2+^ chelating activity	DPPH (93.63 ± 0.13% at 1.0 mg/mL)	LQAEVEELRAALE, IEDPFDQDDWGAWKK, AGRALTAYLMKIL, GYDLGEAEFARIM	[56]
Chickpea seeds	Pepsin, Pancreatin; AC, GFC, nanofiltration	Reducing power assay DPPH radical scavenging activity	-	ALEPDHR, TETWNPNHPEL, FVPH, SAEHGSLH	[57]
Rice residue protein	Papain, Flavourzyme, Protamex; GFC (Sephadex G-15), RP-FPLC	DPPH radical scavenging activity ABTS radical scavenging activity FRAP-Fe^3+^ reducing capacity assay	DPPH (77.6% at 0.5 mg/mL, IC_50_ = 0.25 mg/mL)	RPNYTDA, TSQLLSDQ, TRTGDPFF, NFHPQ	[58]
Rice bran protein	Trypsin; RP-HPLC	ORAC assay	ORAC (4.07 μmol TE/g)	800–2100 Da	[49]
Pinto bean protein isolate	Protamex; UF (100, 50, 30, 10, 3 kDa)	ABTS radical scavenging activity FRAP assay	ABTS (42.2% at 7mM); FRAP (0.81 mM)	PPHMLP, PPMHLP, PLPPHMLP, PLPLHMLP, ACSNHSPLGWRGH, LSSLEMGSLGALFVCM	[59]
Pearl millet	Trypsin; GFC (Sephadex G-25), RP-UFLC	DPPH radical scavenging activity ABTS radical scavenging activity Fe^2+^ chelating activity Reducing power assay ^•^OH scavenging activity	DPPH (67.66% at 1 mg/mL); ABTS (78.81% at 1 mg/mL)	SDRDLLGPNNQYLPK	[60]
*Palmaria palmata* protein	Corolase^®^ PP; SPE, SP-RP-HPLC	ORAC assay, FRAP assay	ORAC (4380.75 ± 66.44 μmol TE/g dw); FRAP (51.86 ± 1.85 μmol TE/g dw)	SDITRPGGQM	[47]
Oat glutelin	Alcalase; IEC, RP-HPLC	^•^OH scavenging activity DPPH radical scavenging activity	^•^OH (IC_50_ = 0.68 mg/mL)	HYNAPAL	[61]
Egg in fresh *Apostichopus japonicus*	Papain, Protamex; UF, HSCCC; GFC (Sephadex G-100, G-50)	^•^OH scavenging activity O_2_^•−^ scavenging activity	^•^OH (93.26, 70.04, and 89.82 U/mL, respectively)	30 kDa (3 kinds of peptides)	[62]
Hazelnut protein	Alcalase; GFC (Sephadex G-25, G-15), RP-HPLC	DPPH radical scavenging activity ABTS radical scavenging activity	DPPH (69.2 ± 1.2%); ABTS (92.9 ± 1.0%)	ADGF, AGGF, AWDPE, DWDPK, ETTL, SGAF	[63]
Pecan protein isolate	Alcalase; UF (10, 5, 3 kDa), IEC, GFC (Sephadex G-50)	DPPH radical scavenging activity ABTS radical scavenging activity ^•^OH scavenging activity Reducing power assay Fe^2+^ chelating activity	DPPH, ABTS, ^•^OH (LAYLQYTDFETR: 56.25%, 67.67%, 47.42% at 0.1 mg/mL)	LAYLQYTDFETR	[23]
Sheep abomasum protein	Papain; UF (10, 3 kDa), IEC; GFC (Sephadex G-50), RP-HPLC	DPPH radical scavenging activity ABTS radical scavenging activity ^•^OH scavenging activity	DPPH (LEDGLK: IC_50_ = 0.63 mg/mL; IDDVLK: IC_50_ = 0.58 mg/mL)	LEDGLK, IDDVLK	[64]
*Erythrina edulis* (pajuro) protein	Alcalase; Polyamide SPE, RP-HPLC	ABTS radical scavenging activity ORAC assay	ABTS (1.37 ± 0.09 μmol TE/mg); ORAC (2.83 ± 0.07 μmol TE/mg)	DGLGYY, CCGDYY, YDLHGY	[65]
Finger millet protein	Trypsin; UF, GFC, RP-UFLC	DPPH radical scavenging activity ABTS radical scavenging activity Fe^2+^ chelating activity ^•^OH scavenging activity	DPPH, ABTS, Fe^2+^ chelating, ^•^OH (61.79%, 78.61%, 51.20%, 66.66% at 1.0 mg/mL)	TSSSLNMAVRGGLTR STTVGLGISMRSASVR	[66]
Cutlassfish muscle	Pepsin; UF, GFC (Sephadex G-25), RP-UFLC	DPPH radical scavenging activity Peroxyl radical scavenging activity	DPPH (IC_50_ = 0.03 mg/mL); Peroxyl (IC_50_ = 0.02 mg/mL)	FSGE	[67]
Sea squirt protein	Pepsin; GFC, RP-HPLC	DPPH radical scavenging activity ABTS radical scavenging activity ORAC assay Reducing power assay Fe^2+^ chelating activity	DPPH (LEW: IC_50_ = 1.29 mM); Fe^2+^ (LEW, MTTL, YYPYQL: 9.20–12.5% at 1 mM)	MTTL, LEW, YYPYQL	[68]
Freeze-dried stone fish flesh	Alcalase; UF, SDS-PAGE, RP-HPLC, Isoelectric point focusing fractionation	DPPH radical scavenging activity ABTS radical scavenging activity FRAP	DPPH (62.5% at 0.1 mg/mL)	GVSGLHID	[69]
Wheat germ protein	Alcalase; RP-HPLC	ABTS	-	TVGGAPAGRIVME, GNPIPREPGQVPAY	[70]
Sesame protein	Alcalase, Trypsin; UF (3, 5, 8, 10 kDa), prep-HPLC	DPPH radical scavenging activity ABTS radical scavenging activity	DPPH (IC_50_ = 2.793 mg/mL); ABTS (IC_50_ = 2.949 mg/mL)	1008.2–1402.7 Da (7 kinds of peptide)	[71]
Sea cucumber collagen	Neutrase; UF (5, 1 kDa) GFC (Sephadex G-15)	DPPH radical scavenging activity ABTS radical scavenging activity	DPPH (35% at 0.2 mg/mL) DPPH (FLAP EC_50_ = 0.385 mg/mL)	FLAP	[72]
Tartary buckwheat albumin	Alkaline Protease; UF (3, 10 kDa), IEC, GFC (Sephadex G-15), RP-HPLC	DPPH radical scavenging activity ^•^OH scavenging activity Reducing power assay Lipid peroxidation inhibition	GEVPW, YMENF, AFYRW: DPPH (IC_50_ = 1.20, 2.91, 0.64 mM); ^•^OH (IC_50_ = 2.21, 1.56, 0.65 mM); Reducing power (0.702, 0.554, 0.927 at 4 mg/mL)	GEVPW, YMENF, AFYRW	[73]
Duck plasma powder	Alcalase; UF (10, 3 kDa), GFC (Sephadex G-25), RP-HPLC	O_2_^•−^ scavenging activity DPPH radical scavenging activity ABTS radical scavenging activity Fe^2+^ chelating activity Reducing capacity	DPPH (88.36% at 1.0 mg/mL) O_2_^•−^ (64.47% at 1.0 mg/mL), ABTS (149.67 mM TE/mg at 1.0 mg/mL)	LDGP, TGVGTK, EVGK, RCLQ, LHDVK, KLGA, AGGVPAG	[74]

^1^ The complete meaning of the abbreviations in the table: ultrafiltration (UF); gel filtration chromatograph (GFC); reverse phase high performance liquid chromatography (RP-HPLC); *2,2*-Diphenyl-*1*-picrylhydrazyl (DPPH); *2,2*-azino-bis (*3*-ethylbenzothiazoline-*6*-sulphonic acid) diammonium salt (ABTS); Oxygen radical absorbance capacity (ORAC); affinity chromatography (AC); ferric reducing antioxidant power (FRAP); reverse phase fast protein liquid chromatography(RP-FPLC); ion exchange chromatography (IEC); hydroxyl Radical (^•^OH); sodium dodecyl sulfate polyacrylamide gel electrophoresis (SDS-PAGE); superoxide anion radical (O_2_^•−^); solid phase extraction (SPE); semi-preparative reverse phase-high performance liquid chromatography (SP-RP-HPLC); reversed phase ultra flow liquid chromatography (RP-UFLC); high-speed countercurrent chromatography (HSCCC); preparative HPLC (prep-HPLC). ^2^ If there is no special label, the antioxidant evaluation data listed here is the data of the peptide fraction with the strongest antioxidant capacity after purification.

**Table 2:** The effect of different pretreatment or co-treatment methods on the preparation of peptidic antioxidants by enzymatic hydrolysis ^1^.

Source	Enzyme	Processing Method	Processing Conditions	Advantages	Ref
Fish frame protein	Alcalase	Microwave	T = 90 °C, t = 5 min	Improved protein solubility, protein recovery, DH, and ABTS radical scavenging activity.	[99]
Barley beer waste protein	Alcalase	Ultrasound	Frequency = 50 Hz, t = 4 h	Improved metal-chelating activity (54%); improved DPPH radical, O_2_^•−^ scavenging, and ^•^OH scavenging activity (28%, 18%, 25%)	[104]
Tilapia by-product protein	Alcalase	High pressure-assisted	Pressure = 250 MPa, t = 35 min	Facilitated the release of low Mw peptides and essential amino acids; improved soluble protein content (5.7 mg/mL), RP (44 μg AAE/g), and solubility (71%) of hydrolysates; decreased IC_50_ (DPPH) values from 653 μg/mL to 304 μg/mL	[106]
Soybean protein isolate	Corolase PP	High hydrostatic pressure	Pressure = 200 MPa, t = 4 h	Enhanced the efficiency of enzymolysis; decreased surface hydrophobicity of hydrolysates; increased the production of small peptides (< 3 kDa); increased RP, ABTS radical scavenging activity	[111]
Egg white protein	Alcalase	Pulsed electric field	Strength = 10 kV cm^−1^, pulsed number = 300, frequency = 3000 Hz	Increased RP ability; broke down larger peptides into smaller peptides	[114]
Pea protein	Papain	Protease co-extrusion	E = 12.0%, T = 60.2 °C, pH = 6.5, S = 7.1%	Enhanced the efficiency of enzymolysis and DPPH radical scavenging activity (98.1%) of enzymatic hydrolysate	[118]
Sweet potato protein	Alcalase, Protease	Radio frequency	T = 80 °C/90 °C	Increased Mw <3 kDa peptide fraction and its antioxidant capacity	[119]
Rice protein	Alcalase	High-energy electron beam	Irradiation doses = 30 kGy	Increased ratio of antioxidative amino acids; produced smaller peptides; increased DPPH and ABTS radical scavenging activity (32.06% and 79.11%) of hydrolysates	[120]

^1^ The complete meaning of the abbreviations in the table: enzyme concentration (E); temperature(T); substrate concentration (S); time (t); reducing power (RP); *2,2*-Diphenyl-*1*-picrylhydrazyl (DPPH); *2,2*-azino-bis (*3*-ethylbenzothiazoline-*6*-sulphonic acid) diammonium salt (ABTS); degree of hydrolysis (DH); superoxide anion radical (O_2_^•−^); hydroxyl radical (^•^OH); molecular weight (Mw).

**Table 3:** Evaluation of in vitro cell model of peptidic antioxidants ^1^.

Source	Peptide	Cellular Model	Cellular Effect	Ref
Whey protein	Hydrophobic peptide	H_2_O_2_-treated PC12 cells	Increased cell survival rate (19.3%); decreased cell death (28.6%)	[212]
Indian squid protein	WCTSVS	H_2_O_2_-treated breast cancer cells (MCF7)	Decreased intracellular ROS	[213]
Soybean protein	FDPAL	H_2_O_2_-treated HeLa cells	Increased cell viability under oxidative stress	[214]
Soybean protein	SHECN	AAPH-treated HepG2 cells	Possessed CAA (776.22 μmol QE/ 100 g)	[215]
Pine nut meal protein	KWFCT, Ac-QWFCT	AAPH-treated HepG2 cells	Possessed CAA (612.8, 916.3 μmol QE/ 100 g)	[216]
Pine nut protein	QDHCH	AAPH-treated/H_2_O_2_-treated HepG2 cells	Possessed CAA (3051.84 μmol QE/100 g); increased SOD, GSH-Px, CAT, GR activities; decreased MDA content increased cell viability under oxidative stress	[217]
Hanwoo beef protein	CCCCSVQK	Human colorectal carcinoma cells (HCT116)	Inhibits the proliferation of HCT116 cells	[211]
Chinese Baijiu	PHP	AAPH-treated HepG2 cells	Increased SOD, GSH-Px, CAT activities; increased GSH content; decreased MDA, GSSG content; decreased intracellular ROS levels	[218]
Rapeseed protein	WDHHAPQLR	H_2_O_2_-treated HUVECs cells	Reduced cell apoptosis	[207]
Perilla seed protein	YL, FY	H_2_O_2_-treated HepG2 cells	Reduced cell apoptosis	[219]
Lupin protein confer	Peptides with Mw < 3 kDa	H_2_O_2_-treated HepG2 cells	Increased cell survival rate; decreased intracellular ROS levels; increased SOD, GSH-Px	[220]
Soybean protein	IYVVDLR; IYVFVR, VVFVDRL, VIYVVDLR	H_2_O_2_-treated Caco-2 cells	Increased CAT, GR activity (IYVVDLR, IYVFVR); increased GSH content (IYVVDLR, IYVFVR, VVFVDRL); increased cell viability under oxidative stress (IYVVDLR, IYVFVR, VVFVDRL); decreased MDA content; decreased intracellular ROS levels	[221]
Fermented grain (Jiupei)	VNP, YGD	AAPH-treated HepG2 cells	Increased SOD, GSH-Px, CAT activities; decreased intracellular ROS levels; decreased MDA, GSSG content; increased GSH content	[222]
Defatted walnut meal	VEGNLQVLRPR, LAGNPHQQQQN, HNLDTQTESDV, AGNDGFEYVTLK, QQRQQQGL, AELQVVDHLGQTV, EQEEEESTGRMK, WSVWEQELEDR	H_2_O_2_-treated SHSY5Y cells	Decreased intracellular ROS levels (ex WSVWEQELEDR); increased cell viability under oxidative stress	[223]
Mulberry leaf protein	SVL, EAVQ, RDY	AAPH-treated HepG2 cells	Possessed CAA (1706, 1501, 2204 μmol QE/ 100 g); inhibited oxidant-induced hemolysis (RDY: 92%)	[224]
Egg white protein	VYLPR	H_2_O_2_-treated HEK-293 cells	Increased cell viability under oxidative stress (97.45%); increased SOD, GSH-Px activities; decreased MDA; inhibit LDH activity	[225]
Collagen from sea cucumber	Peptides with Mw < 1 kDa	H_2_O_2_-treated RAW264.7 cells	Promote cell proliferation; decreased intracellular ROS levels; decreased intracellular ROS levels; increased SOD, GSH-Px activities; decreased MDA	[226]
Collagen of Redlip Croaker	GPEGPMGLE, EGPFGPEG, GFIGPTE	H_2_O_2_-treated HepG2 cells	Decreased intracellular ROS levels; decreased MDA; increased SOD, GSH-Px, CAT activities	[227]
Fermented milk	NTVPAKSCQAQPTTM, EDELQDKIHPF, QGPIVLNPWDQVKR, APSFSDIPNPIGSENSE	T-BHP-treated Caco-2 cells	Increased cell viability under oxidative stress; decreased intracellular ROS levels	[129]
Whey protein	Peptides with Mw ≤ 3 kDa	Menadione-treated IEC-18 cells	Increased cell viability under oxidative stress (88%)	[228]

^1^ The complete meaning of the abbreviations in the table: cellular antioxidant activity (CAA); lipophilic tert-butyl hydroperoxide (*t*-BHP), water-soluble *2,2*-azobis-(*2*-amidinopropane) dihydrochloride (AAPH); reactive oxygen species (ROS); glutathione peroxidase (GSH-Px); malondialdehyde (MDA); superoxide dismutase (SOD); catalase (CAT); oxidized glutathione (GSSG); glutathione (GSH); molecular weight (Mw).
